# Multicriteria plan optimization in the hands of physicians: a pilot study in prostate cancer and brain tumors

**DOI:** 10.1186/s13014-017-0903-z

**Published:** 2017-11-06

**Authors:** Birgit S. Müller, Helen A. Shih, Jason A. Efstathiou, Thomas Bortfeld, David Craft

**Affiliations:** 1 Department of Radiation Oncology, Massachusetts General Hospital, Harvard Medical School, Boston, MA USA; 2 Department of Radiation Oncology, Klinikum rechts der Isar, Technical University of Munich, Ismaninger Straße 22, 81675 Munich, Germany; 3 Department of Physics, Technical University of Munich, Munich, Germany

**Keywords:** IMRT, Multicriteria optimization, Efficiency, Physician IMRT planning

## Abstract

**Background:**

The purpose of this study was to demonstrate the feasibility of physician driven planning in intensity modulated radiotherapy (IMRT) with a multicriteria optimization (MCO) treatment planning system and template based plan optimization. Exploiting the full planning potential of MCO navigation, this alternative planning approach intends to improve planning efficiency and individual plan quality.

**Methods:**

Planning was retrospectively performed on 12 brain tumor and 10 post-prostatectomy prostate patients previously treated with MCO-IMRT. For each patient, physicians were provided with a template-based generated Pareto surface of optimal plans to navigate, using the beam angles from the original clinical plans. We compared physician generated plans to clinically delivered plans (created by dosimetrists) in terms of dosimetric differences, physician preferences and planning times.

**Results:**

Plan qualities were similar, however physician generated and clinical plans differed in the prioritization of clinical goals. Physician derived prostate plans showed significantly better sparing of the high dose rectum and bladder regions (p(D1) < 0.05; D1: dose received by 1% of the corresponding structure). Physicians’ brain tumor plans indicated higher doses for targets and brainstem (p(D1) < 0.05). Within blinded plan comparisons physicians preferred the clinical plans more often (brain: 6:3 out of 12, prostate: 2:6 out of 10) (not statistically significant). While times of physician involvement were comparable for prostate planning, the new workflow reduced the average involved time for brain cases by 30%. Planner times were reduced for all cases. Subjective benefits, such as a better understanding of planning situations, were observed by clinicians through the insight into plan optimization and experiencing dosimetric trade-offs.

**Conclusions:**

We introduce physician driven planning with MCO for brain and prostate tumors as a feasible planning workflow. The proposed approach standardizes the planning process by utilizing site specific templates and integrates physicians more tightly into treatment planning. Physicians’ navigated plan qualities were comparable to the clinical plans. Given the reduction of planning time of the planner and the equal or lower planning time of physicians, this approach has the potential to improve departmental efficiencies.

## Background

The creation of a radiotherapy treatment plan is a stepwise process involving a diverse mix of staff. In clinical practice treatment plans are usually generated by dosimetrists or medical physicists^1^, translating written clinical prescriptions into dose distributions. The treatment plan optimization is a multicriterial problem which leads to inevitable trade-offs between targets and organs at risk (OAR) such that not all clinical goals can always be fulfilled [1–3]. Potential dosimetric conflicts might not be obvious before the initiation of planning and thus might not be addressed in the prescription. Clinical decisions have to be made during planning as they arise. Often planners^1^ spend much time trying to find a compromise between the different clinical goals, which might not be the trade-offs most preferred by the physician. When plans are presented to the physicians for clinical approval, it is often the first time the physician has the opportunity to review the dose distribution and many intermediate decision points with minimal or no physician input may have been made.

Many physicians accept a plan if it fulfills the prescribed dosimetric goals but it may not represent the most suitable compromise between different objectives. Being aware of the possibilities and weighing associated clinical consequences physicians might select a different trade-off. Making those clinical trade-off decisions should ultimately be in the hands of the staff trained to make them, i.e. the physicians.

Moreover, this current clinical planning practice frequently results in iterative plan adaptations until the plan is approved by the physician – a time consuming process for both physician and planner.

In addition to the interaction between physicians and planners for clinical decisions, the actual planning is frequently inefficient. Planning is often a trial-and-error process, with the quality of the final plan dependent on the skills or personal perception of the planner. Planners use different helper structures and parameters based on experience and knowledge. Many attempts to standardize treatment planning and improve its consistency by finding class solutions and using knowledge based planning have been reported [4–9].

Multicriteria optimization (MCO) has proven to be an efficient treatment planning method, both in terms of planning time and dosimetric quality [1, 10–12]. It is applicable to problems where there is no single clear optimal solution, but instead the problem requires compromises. These compromises are described mathematically on the so-called Pareto surface of optimal plans [13, 14]. Plans on the surface cannot be improved in one criterion without worsening another [13, 15]. MCO planning software eliminates the time-consuming trial-and-error process of selecting suitable weighting factors in conventional IMRT planning [16–19] and visualizes these dosimetric trade-offs. It provides a tool to interactively navigate on the surface and investigate the trade-offs [16, 17] which makes planning more intuitive but yet may not be fully exploited in the current clinical planning routine.

One approach to make use of the MCO system, and to improve treatment planning, both in achieving the desired dosimetric goals and in increasing efficiency, is to involve the physician at an earlier stage of the planning procedure.

We suggest physician driven planning as an alternative planning procedure, which - similar to the current workflow - consists of a collaboration of physicians and planners, but differs in the order and responsibility of involved tasks. Physician driven planning utilizing MCO treatment planning software avoids the “human iteration loop” between physicians and planners by providing physicians the control over trade-offs, and allows them to tailor the treatment plans to the individual patient.

**Fig. 1:**
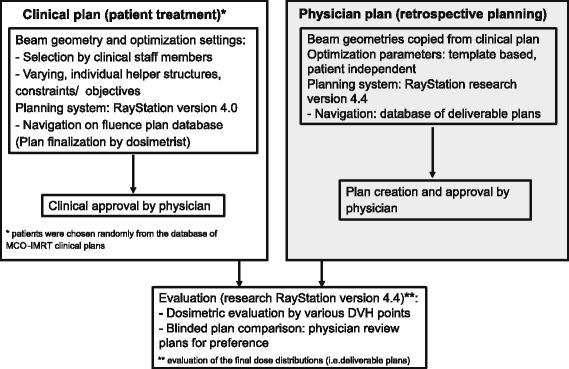
Planning study workflow

In a retrospective planning study, we demonstrate the feasibility of physician driven planning by template based optimization and physician plan navigation as a suitable planning procedure, and assess whether it has the potential to improve planning efficiency and quality. We compare clinically delivered plans, created by dosimetrists, to plans that physicians interactively navigated (Fig. 1).

## Methods

The study is based on data from 12 brain tumor and 10 prostate cancer patients previously treated with MCO optimized intensity modulated radiotherapy (IMRT) (step and shoot) at Massachusetts General Hospital (MGH) and selected randomly out of the clinical database.

### Structure definition

#### Brain tumors

The patients featured a variety of diagnoses and intracranial anatomical sites. The gross tumor volume (GTV) was defined based on integrated CT and magnetic resonance imaging from preoperative and postoperative studies. Clinical target volumes (CTV) were created to encompass additional regions of potential microscopic involvement. Prescriptions to the planning target volume (PTV) (CTV + 3 mm) ranged from 36 Gy (12 × 3 Gy) to 60 Gy (30 × 2 Gy). Standard OARs defined for all cases included the brainstem, chiasm, optic nerves, eyes, lenses, lacrimal glands, and cochleae. Tolerance doses varied dependent on the individual case, i.e. tumor location and prescribed dose. For 60 Gy commonly given constraints were amongst others: maximum dose D_max_(optic nerves and chiasm) < 54 Gy, D_max_(orbits) < 45 Gy, D_max_(brainstem) < 54 Gy in center, < 60 Gy on the surface, and a maximum of 57 Gy to 5% of the volume.

#### Prostate cancer

All cases had undergone radical prostatectomy and were receiving postoperative radiation therapy. The CTV (prostatic fossa), PTV, rectum, bladder (excluding CTV) and femoral heads were contoured for all patients. Small bowel, sigmoid, residual seminal vessels and penile bulb were added dependent on individual patients’ anatomy and clinical situation. The prescribed dose to the PTV (CTV + 8 mm, posterior: + 4 mm) was 66.6 Gy (37 × 1.8 Gy). For other planning parameters see Table 1.

**Table 1:** Typical MCO-problem formulations for prostate (prescription: 66.6 Gy); EUD: equivalent uniform dose [29], *excluding PTV wall

Constraints		Objectives (slider)	
PTV	Min/ max dose = prescribed dose +/− 15%	PTV	1) Min dose = prescribed dose
			2) Max dose = prescribed dose
CTV	Min DVH: prescribed dose to 95% volume	CTV	Min dose = prescribed dose
PTV wall (1 cm ring around PTV)	Max dose = prescribed dose +3%		
Rectum/ bladder (excluding CTV)	Max dose = prescribed dose +5%	Rectum/ bladder (excluding CTV)	Dose-fall off: prescribed dose to 0 Gy in 1 cm
Normal tissue*	Max dose = prescribed dose	Normal tissue	Dose-fall off: prescribed dose to 0 Gy in 1 cm
		Femurs	EUD, a = 2

### MCO treatment planning

**Fig. 2:**
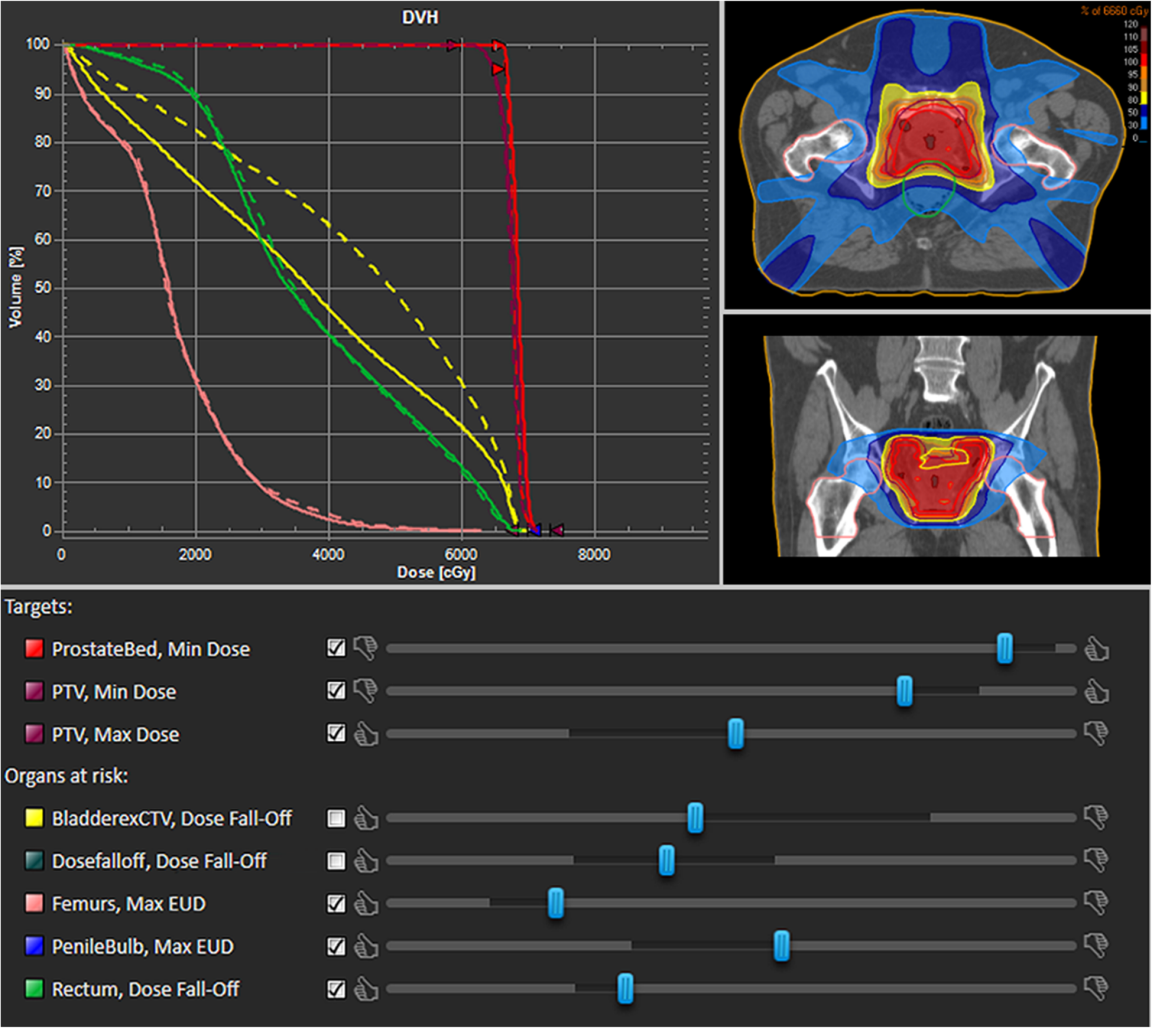
Interface components of MCO-plan navigation: dose distributions are adjusted in real time by moving the sliders of different structures. The dosimetric changes are also visualized in the dose volume histogram: current (straight line) and previous (dashed line) dose

MCO-planning comprises the Pareto front calculation, the subsequent plan navigation and deliverable plan creation. The Pareto front is calculated by optimizing various weighted sums of prior defined treatment objectives. For n objectives at least n + 1 plans are calculated [15], and the maximum plan number is given by the software with approximately 4n (default usage for the study). Each treatment objective is represented in the treatment planning system (TPS) navigation interface by a slider (Fig. 2). It improves the corresponding objective function and updates the dose distribution in real time when moved by interpolating between pre-computed plans. After navigation, plans are finalized by multileaf collimator (MLC) sequencing and final dose calculation.

Clinical plans were generated in the commercial clinically implemented MCO-planning system RayStation (version 4.0; RaySearch, Stockholm, Sweden); retrospective physician driven plans were created in a research software version (research version 4.4) (Fig. 1). Whereas plan navigation in the commercial software is based on fluence maps, the research module allows for navigation on segmented plans which decreases dose differences of the final planning step [20, 21] and may therefore be more suitable for physician driven planning. With regards to all other planning and dosimetric parameters, including the dose calculation, the planning systems are identical.

### Planning and optimization parameters

The clinically chosen beam settings were also used for retrospective physician planning (prostate: 7 beams, brain tumors: 4–7 beams) to ensure that all found dose differences between the clinically and physician generated plans were the result of user preferences of plan nuances rather than fundamental factors such as beam direction. The clinical plans that served as our baseline comparison plans were made by the clinical dosimetry treatment planning staff who did not know that their plans would be used in a retrospective comparison study. At MGH, treatment site specific optimization templates exist and are recommended for use. As planners often change these as they wish, clinically utilized planning helper structures and optimization parameters varied between planners and patients. For the retrospective planning study a database of Pareto optimal plans was created using mostly patient independent but site specific, self-developed, templates (Table 1) in order to test the idea that a template based Pareto-surface creation technique was suitable for physician-based MCO planning. The set of constraints contained loose minimum and maximum doses to prevent extreme under- and overdosage in the target and uncompromising doses to OARs, but not to restrain physicians in their options. A detailed explanation of the implemented objective functions can be found in [22].

### Physician plan navigation

Physicians, who treated these patients before, were provided with the anonymized patients, the plan database and patient information (prescription, constraints and history); no access was given to the prior clinically treated plan. Prostate plan navigation (Fig. 2) was conducted with 9 sliders on average (max.10), brain tumor cases with 15 (max. 16), amongst those a maximum of three sliders for the target (two for the PTV, one for CTV or GTV) (Table 1).

### Plan evaluations and efficiency analysis

The final clinical and physician dose distributions were analyzed by several dose volume histogram (DVH) values. Statistical evaluations were performed by paired t-tests and non-parametric signed rank tests. Two weeks after planning physicians were asked for their plan preference, for each patient case, in a blinded comparison. Preferences were rated as slightly or significantly different. The option “no preference” referred to equal plan quality.

**Fig. 3:**
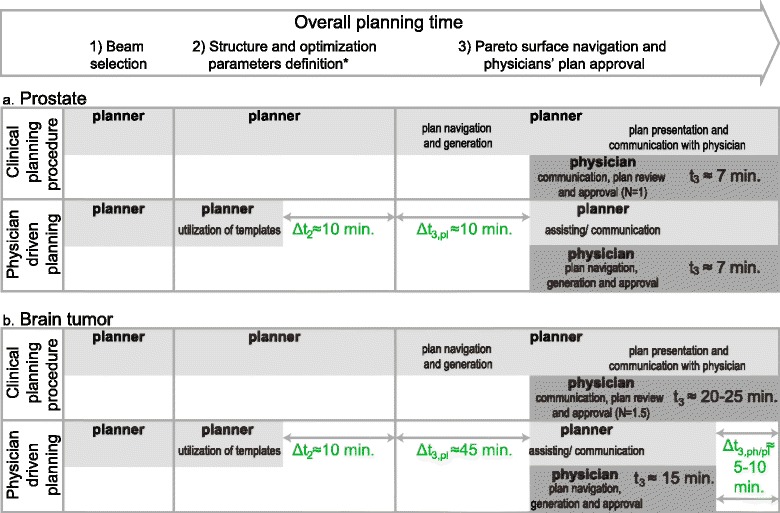
Comparison of planning efficiency of current clinical planning versus physician driven planning of prostate (**a**) and brain tumor cases (**b**). * refers to the selection and – where applicable - creation of helper structures for the optimization process

We analyzed required planning times of planners and physicians for both procedures in a theoretical comparison of the main components, i.e. 1) beam selection (time t_1_), 2) selection plus creation of helper structures for the optimization (where applicable) and Pareto surface calculation (t_2_) and 3) navigation plus physician approval (t_3_). Planners are involved in all steps (t_planner_ = t_1_ + t_2_ + t_3,pl_), physicians exclusively in the last step (t_ph_ = t_3,ph_) (Fig. 3). Physicians’ navigation times, including the deliverable plan generation, were recorded, and clinical times were determined based on departmental interviews of staff members. In clinical planning (CP), the required times are driven by the number of iterations N between both staff members until a plan is approved by the physician. Average physician and planner times t_3,ph_(CP) and t_3,pl_(CP) were calculated by the time to coordinate between physician and planner, including the communication of new instructions, to review and approve the plan, multiplied by the number of iterations of plan adaptations before approval.

## Results

### Dosimetric differences

**Fig. 4:**
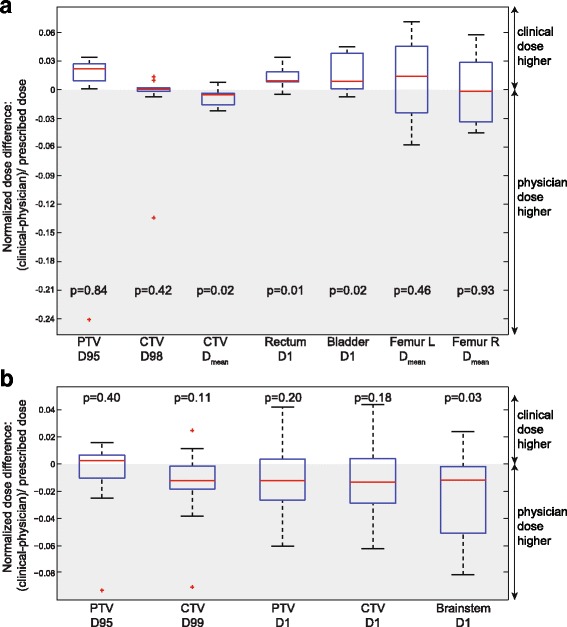
**a** Normalized dose differences of prostate plans of performed t-tests; we also computed the non-parametric Wilcoxon signed rank p-statistics since some of the difference data do not pass the Lilliefors normality test (due to the outliers). Those *p*-values lead to the same conclusions as the p-values from the t-test. Starting with PTV D95, they are 0.08, 0.70, 0.03, 0.004, 0.04, 0.43, and 0.92. **b** Normalized dose differences of brain tumor plans of performed t-tests; p-values of the Wilcoxon signed rank test, starting at PTV D95, are 0.97, 0.05, 0.23, 0.20, 0.019; remarks: CTV refers to 11 CTVs and one GTV contour; brainstem statistics based on 11 contours

Most evaluated criteria did not show significant differences or trends but were spread around zero, D_mean_ of the femoral heads (Fig. 4a) being a typical example. Prostate plans, generated by the physician, showed a better sparing of high dose regions of bladder and rectum, as demonstrated by the significant differences in V65(rectum) (*p* = 0.009) and V65(bladder) (*p* = 0.003) (relative volume receiving at least 65 Gy). The PTV covered by the 98%-isodose was significantly lower (V65 (PTV): *p* = 0.007) while the CTV received higher mean doses. The trend of greater PTV-coverage in clinical plans is presented in Fig. 4a by D95(PTV) (dose covering 95% of the PTV). One outlier did not follow this trend but showed a low D95(PTV) (due to small bowel sparing). Ignoring this plan in the statistical evaluations would lead to significance for D95(PTV) with *p* < 5% while the trend of all other criteria would be the same.

**Fig. 5:**
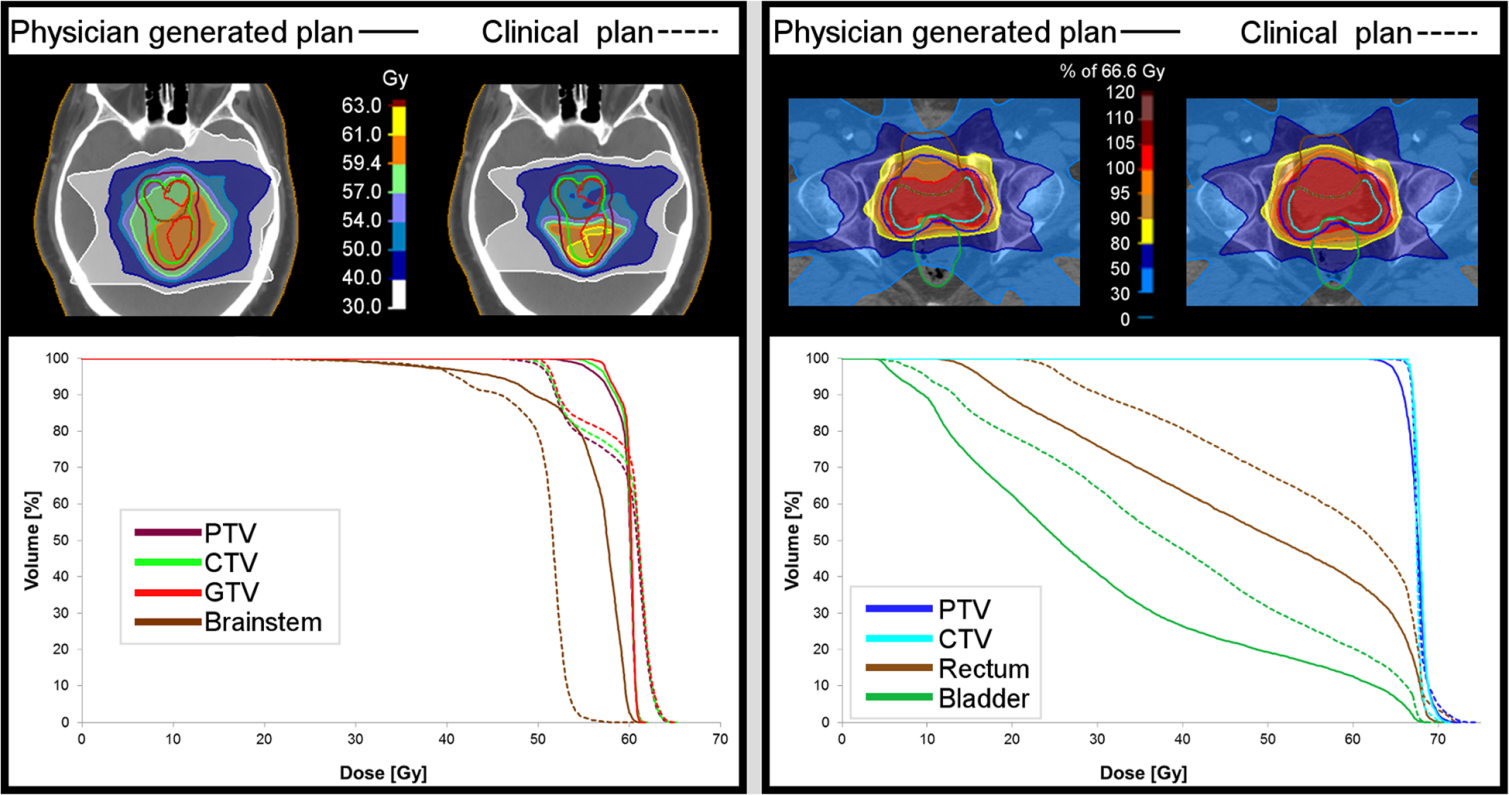
Case examples of remarkable differences between clinical and physician planning for brain tumor and prostate; blinded plan comparison resulted in "no preference" for brain tumor (physician preferred an average of both) and in favor of physician prostate plan

Physician derived brain tumor plans indicated a trend to higher doses in the targets and OARs, with a significantly higher maximum dose to the brainstem (D1(brainstem): *p* = 0.03) (Fig. 4b). Dose distributions of one brain tumor and one prostate example showing clear dosimetric differences are shown in Fig. 5.

**Fig. 6:**
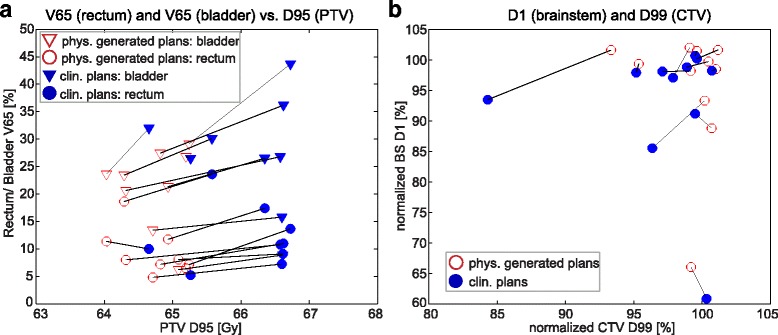
Dosimetric trade-offs of selective DVH criteria for prostate (**a**) and brain tumor plans (**b**). Each data point represents the compromise between two dosimetric objectives of one plan; physician (phys.) generated plan results are linked to corresponding clinical (clin.) plan trade-offs. Nine prostate and 11 brain tumor cases are presented (excluding the prostate outlier, see Fig. 4a, and the brain case without brainstem contour). (note: plans which are superior in all presented DVH criteria than the corresponding plan were worse in at least one other dosimetric objective that is not included in the figure)

Trade-off plots of different dosimetric objectives present the chosen compromises by physicians and dosimetrists (Fig. 6). Except for the navigation to higher CTV doses for a cost in increased D1(brainstem) (Fig. 6b), no comprehensive trade-offs over all patients were found for brain tumor plans; chosen compromises differed between patients.

### Physicians’ preferences

The blinded plan comparison results of physicians’ preference are presented in Table 2, which shows a slight preference for the clinically generated plans. The doctors decided all plans, whether selected or not, were clinically acceptable. However, for one brain tumor case (Fig. 5) the physician would have preferred to navigate again in order to achieve the average of the presented plans. Prostate plans were additionally rated by a non-planning involved physician who voted in favor of physician plans.

**Table 2:** Physicians’ plan preferences: results of blinded plan comparison; *physician 1 = planning physician, physician 2 = a non-planning involved physician

	Brain tumors		Prostate (physician 1*)		Prostate (physician 2*)	
	Preference	Degree	Preference	Degree	Preference	Degree
Physician generated plan	3	all slightly	2	1 slightly, 1 significantly	5	3 slightly, 2 significantly
Clinical plan	6	5 slightly, 1 significantly	6	4 slightly, 2 significantly	3	2 slightly, 1 significantly
No preference	3	–	2	–	2	–
Total	12	–	10	–	10	–

### Physicians’ experiences

On a more qualitative basis, recorded physicians’ statements during the sliding sessions, such as: “it is interesting how this dose increases while the other one decreases” or “that was a good deal” demonstrated the physicians appreciating having the control over the dose. Experiencing dosimetric trade-offs improved their comprehension of the planning situations.

### Planning efficiency

#### Planners’ time

The first planning step, the beam selection, is identical for both planning approaches (Fig. 3). In the second step physician driven planning reduces planners’ time through the utilization of pre-defined optimization templates, which can save up to Δt_2_ = 10 min, the approximate average time it takes to decide on objectives and constraints and add them to the formulation. For step 3, in clinical planning, the required number of iterations N until a plan is approved (see above) is determined to be *N* = 1 for prostate planning, as it is a standardized procedure and plans are mostly accepted at the first review. For brain tumor cases N varies between one, two, and occasionally three. We assume an average of *N* = 1.5 per plan. Physician driven planning eliminates planners’ navigation completely which corresponds to a time of approx. 10 min in simpler cases (see e.g. [1]) and up to 30 min in complex cases. Considering the factors of 1.0 and 1.5 (iterations), planner time Δt_3,pl_ decreases by 10 and 45 min for prostate (Fig. 3a) and brain cases (Fig. 3b), respectively.

#### Physician times

Clinical physician times to coordinate and communicate with planners are on average 2 min for prostate and 5 min for brain cases. Plan review and approval take approximately 5 min for prostate and 10 min for brain cases (complexity comparable to lung tumors, average times published by [23]). Taking the number of iterations into account, physician times were estimated by 7 min for prostate (N = 1; t_ph_(CP) = t_3,ph_(CP) ≅ 2 + 5 min) and 20–25 min for brain cases (N = 1.5; t_ph_(CP) = t_3,ph_(CP) ≅ 1.5 × 15 min).

Average physician navigation times were 10 min for prostate and 16 min for brain tumor. Distinguishing average times by the first and second half of navigated plans, times decreased from 13 to 7 min (averages over 5 plans) and 17 to 15 min (averages over 6 plans) for prostate and brain tumors, respectively.

The achieved physician time savings between both approaches were calculated by subtracting the average navigation time (after more training) from the clinical involvement times, which results in no time difference for prostate (t_ph_(PP) = t_3,ph_(PP) ≅ 7 min) (Fig. 3a) and between 5 and 10 min for brain tumor cases (t_ph_(PP) = t_3,ph_(PP) ≅ 15 min) (Fig. 3b), corresponding to a reduction of 30% of the involved time.

## Discussion

We presented a planning approach with physician navigation as a defining part of the process. While making planning more patient independent by creating standardized databases, our approach makes it more individualized for each patient by not following the same standard prescription for everyone but finding the best trade-offs for every individual. This study sought to determine if standardized MCO templates plus physician navigation of the resulting Pareto surfaces could offer a viable alternative to the standard planning process, where tradeoffs are explored by the treatment planners and the physicians are only involved in the final YES/NO decision.

Our retrospective planning study is subject to limitations given by inherent differences between clinical and physician driven planning. Although clinical plans were generated by different dosimetrists, they were regarded as comparable. Different qualities within plans may have resulted due to individual planning strategies and personal preferences. The utilization of different TPS software versions may have led to a slight advantage in the navigation process for physicians. As dosimetrists are well trained to the clinical software version, and as the intention of the study was not to compare minor but rather fundamental dose differences, this difference is assumed not to have an impact on the study results (for details on dosimetric differences between the two software versions see [21]). Further limitations are found in the restricted number and variety of cases and of participating planning physicians (one for each case).

As the study was performed retrospectively, the exact clinical planning times were not recorded, which prohibited a plan by plan comparison of required times for each step of the procedure. Instead we reverted to clinical experiences of staff members and estimated average times. Commonly, physician times are scarce due to their clinical schedules, and the most expensive. This motivates the aim to shorten physician involvement by utilizing their time as best as possible. While for prostate cases, required times to navigate were comparable to the times to review and approve plans, physician driven planning achieved sizeable time reductions for brain tumors. Compared to prostate planning, these more complex cases are usually subject to more dosimetric conflicts and require more communication. Figure 5 presents a brain tumor case for which the physician prioritized better target coverage over the hard constraint on the brainstem. While dosimetrists usually respect formulated hard constraints, physicians will not always rigidly adhere to their standard prescriptions when compromises are required. Not only in cases of incompatible goals but also when all constraints are achievable simultaneously, physician background knowledge is important in order to decide how best to distribute or escalate the dose.

Contrary to possible expectations, the blinded comparison showed a slight preference (not statistically significant) for the clinical plans for both physicians. In our view, this reflects the fact that the treatment planners 1) are experienced and 2) have effective working relationships with the physicians such that they can achieve plans deemed desirable by both physicians. In light of these ideas, we find it encouraging from the perspectives of increased throughput and increased physician involvement that the plan quality of both planner and physician generated plans are on par. Picking a preference between two plan options was difficult. To examine whether the preference decisions were stable, prostate plans were rated by a second non-planning involved physician. While the planning physician voted 2:6 for the physician vs. the clinical plan (Table 2), the second doctor voted 5:3, favoring the physician plans. Both physicians chose equally good twice but on different cases.

The choice of preferences demonstrated the inter-observer variability [24] of opinions on plan quality. Generally, plan preferences are considered to be caused by personal clinical experiences, e.g. having recently experienced certain patient side-effects might lead to a different point of view. Having a second physician review the cases highlighted the result from the initial physician selections that the two plans for each case are both acceptable plans.

Due to the ongoing learning process of physicians throughout the study, a final conclusion on physicians’ dose preferences might be too early and would require a planning study with more physicians at a later stage of MCO-training. This feasibility study was performed with minimal trained physicians to demonstrate that physicians can do plan navigation. Further improvements of plan qualities are expected by improving optimization formulations, e.g. by adapting objective functions and structures to physicians’ needs instead of dictating them.

Generally, the choice of optimization parameters is crucial. Translating physicians’ desires for each organ by one slider is challenging and not always possible, given that judging the dose distribution on even a single organ is a multi-dimensional task [10]. In general it is difficult to steer three dimensional dose distributions by using one criterion, e.g. trying to get rid of a hot spot at a certain position might reduce dose of that structure at a different location simultaneously. The development of objective functions that allow for a more accurate and specific steering and control of the doses could improve planning in the future.

Our study provided insight into the planning process to physicians with which they are often not familiar. Involving physicians is not only feasible but can also be regarded as a gain of knowledge on more levels. Knowing the expense of a certain dosimetric goal like a homogeneous target dose distribution might change the physicians’ point of view or expectations to a plan [25]. Even if clinical planning and navigation is not done by the physicians it is a gain for the physician to understand the process, and experience the trade-offs also for comprehending difficulties planners sometimes face.

MCO-treatment planning could also serve as a tool for educational purposes and in clinicians’ rounds. Being aware of realizable dose distributions, physicians could better specify the prescriptions and clinical goals of each individual case [26]. Clearer formulated prescriptions will shorten the “back and forth” process between planner and physician and thus make the planning process more efficient.

The suggested procedure may serve as a basis for a discussion to refine optimal planning workflow. Obviously the planning cannot be done by physicians only, as the whole plan creation would be too time consuming for an efficient clinical workflow and best utilization of clinician time. Here physicians were provided with the prepared database, including prior required work steps such as beam angle selections, and physician involvement consisted of the final plan navigation and generation. The whole planning process is and will stay an interactive process of a mix of professions. Our study did not demonstrate the general superiority of this approach, but indicated that deviation from the standard procedure has the potential to improve planning efficiency, and possibly quality (physician navigation skills improved over time). Finding the “best” approach to this interactive process of different professions, in terms of efficiency and resulting quality, still requires further investigations.

It could be promising to combine MCO with knowledge-based planning [7–9, 27, 28]. While automated knowledge-based planning can generate good plans that are sometimes even preferable to planner-generated IMRT plans, physicians still need to apply clinical judgment to make the best treatment decision for each individual patient. In our experience, the best way to accomplish this is through the use of MCO – except for the most trivial cases.

Knowledge based planning and MCO are both needed and they are synergistic: the knowledge-based system could provide the templates and beam orientations, as well as the starting point for the interactive navigation. The physician would then be involved in making relatively minor adjustments using only a small number of sliders/trade-offs, without being overwhelmed by too many options.

We believe that planning systems of the future will be both knowledge-based and MCO-based.

## Conclusion

We demonstrated the feasibility of physician driven planning in MCO treatment planning by Pareto surface navigation on template based optimized plans. The plan quality of physician generated plans was comparable to the clinical plans with differences being observed due to focusing on different clinical goals. This workflow always reduces the required planner time while the reduction in physician time is case dependent and potentially greater for more complex cases. Although the evidence for superior plan quality was not proven within this study, and is certainly also case and patient dependent, it demonstrated that increasing physician involvement and at an earlier stage into the planning process is feasible and has the potential to improve departmental efficiency.

## Abbreviations

CTV: Clinical target volume
GTV: Gross tumor volume
IMRT: Intensity modulated radiotherapy
MCO: Multicriteria optimization
MGH: Massachusetts General Hospital
MLC: Multileaf collimator
OAR: Organ at risk
PTV: Planning target volume
TPS: Treatment planning system

## Footnotes

1 planning personnel varies between countries and clinics; in the following “planner” refers to both professions.
